# Biodegradable Cassava Starch/Phosphorite/Citric Acid Based Hydrogel for Slow Release of Phosphorus: *In Vitro* Study

**DOI:** 10.3390/gels10070431

**Published:** 2024-06-28

**Authors:** Andrés F. Chamorro, Manuel Palencia, Enrique M. Combatt

**Affiliations:** 1 Research Group of Electrochemistry and Environment (GIEMA), Faculty of Basic Sciences, Universidad Santiago de Cali, Cali 760035, Colombia; 2 Research Group in Science with Technological Applications (GICAT), Department of Chemistry, Faculty of Natural and Exact Science, Universidad del Valle, Cali 760032, Colombia; 3 Department of Agricultural and Rural Development, Faculty of Agricultural Sciences, Universidad de Córdoba, Monteria 230002, Colombia; ecombatt@fca.edu.co

**Keywords:** hydrogels, Cassava starch, P slow release, phosphorite

## Abstract

Phosphorous (P) is one the most important elements in several biological cycles, and is a fundamental component of soil, plants and living organisms. P has a low mobility and is quickly adsorbed on clayey soils, limiting its availability and absorption by plants. Here, biodegradable hydrogels based on Cassava starch crosslinked with citric acid (CA) were made and loaded with KH_2_PO_4_ and phosphorite to promote the slow release of phosphorus, the storing of water, and the reduction in P requirements during fertilization operations. Crosslinking as a function of CA concentrations was investigated by ATR-FTIR and TGA. The water absorption capacity (WAC) and P release, under different humic acid concentration regimens, were studied by in vitro tests. It is concluded that hydrogel formed from 10% *w*/*w* of CA showed the lowest WAC because of a high crosslinking degree. Hydrogel containing 10% *w*/*w* of phosphorite was shown to be useful to encouraging the slow release of P, its release behavior being fitted to the Higuchi kinetics model. In addition, P release increased as humic acid contents were increased. These findings suggest that these hydrogels could be used for encouraging P slow release during crop production.

## 1. Introduction

The rapidly growing world population demands an increase in food production to ensure food and nutritional security. In this context, in recent decades, there has been a growing demand for resources in the agricultural field, which has placed pressure on ecosystem resources such as water and soil. In the first case, as the agricultural frontier increases, water requirements are increasing as well, which competes with the supply needs of the community, fauna, and other productive sectors. On the other hand, due to over-exploitation, the productive capacity of soils is diminished, experiencing a marked decrease in their agricultural suitability. The above is usually addressed through the increasing incorporation of fertilizers, mainly nitrogen (N), phosphorus (P) and potassium (K), which in turn, when used excessively and in an uncontrolled manner, negatively impact the environment by disturbing ecosystem balances, through processes such as eutrophication, and the progressive degradation of the physical, chemical, and biological properties of soils (i.e., salinization, erosion, loss of structure, disintegration and loss of the clay fraction, decrease in the population of microorganisms, etc.). For example, chemical fertilizers can cause soil acidification, increasing the availability of heavy metals such as Cd found in phosphate fertilizers derived from phosphate rock [1]. Another example is the salinization and disintegration of the soil structure due to the increase in salts, such as sodium and potassium, which increase the soil pH and promote erosion; but also, water availability for plants is reduced [2]. In addition, hazardous emissions resulting from the hydrolysis of nutrients and the action of microorganisms (i.e., NH_3_, N_2_O, etc.), residue accumulation, and pesticide resistance in insects and pathogens pose risks to human health, among other concerns [3,4,5,6].

As consequence, agricultural-use products including fungicides, herbicides, nematicides, fertilizer, and other substances are used in large quantities [7]. Particularly, though fertilizers improve the crop production, 60–90% of them are lost due to evaporation, adsorption, degradation, and environmental runoff [8,9]; consequently, the costs of crop production are increased and transferred to the final consumer, restricting its accessibility in the most impoverished communities. In this way, accessibility to food is restricted for the population with lower purchasing power. As a result of the above, new technologies focused on improving the supply of fertilizers, increasing their use, and reducing their losses are highly desired.

Polymer-based controlled-release systems are a technological alternative set out to overcome the disadvantages and side effects of the uncontrolled (or inadequate) use of fertilizers. Recently, the utilization of nano- and micromaterials has been a strong alternative set out to promote the slow delivery of fertilizer to plants [10]. However, whereas slow-release fertilizers are usually prepared by the encapsulating of urea, used as nitrogen source, slow-release systems based on the encapsulation of phosphorus and potassium are limited. Furthermore, the agricultural application and industrialization of nanomaterials are still limited due to limited information on the human and environmental health risks of them; but also, there are technical and scientific reports that show the regulatory difficulties resulting from the uncertainty of the dynamics of nanomaterials when they are released into uncontrolled environments [11]. For instance, ZnO–urea nanoparticles reduce N leaching in the soil because they can be taken up by plant roots, decreasing fertilizer loss. However, they produce oxidative stress in soybean roots, affecting hormone metabolism [12]. Additionally, ZnO nanoparticles cause death in aquatic organism such as *Daphnia magna*, *Lymnaea stagnalis*, *Caenorhabditis elegans* [13], and *P. lividus embryos* [14,15]. This negative effect has also been observed in human cells, where ZnO nanoparticles decrease cell viability, produce DNA damage, and cause carcinogenesis [16]. In humans, medical studies on ZnO nanoparticles are limited, but there are reports of severe pulmonary inflammatory diseases [17]. Another limitation is the intrinsic complexity of the characterization and standardization of its properties during the large-scale manufacturing process, which implies the use of technology the costs of which are transferred to the product, leading to a higher relative cost of these compared to other technologies. On the contrary, materials on the microscale are easier to manufacture, control, characterize and standardize, and consequently both their properties as well as their effects have less uncertainty, which implies lower risks.

On the other hand, eco-friendly hydrogels are highly hydrophilic polymeric materials with the ability to absorb a large amount of water. Interest in these materials for application in agriculture has increased because of their ability to store and release both water and the solutes dissolved in it; therefore, they can be used to release fertilizers and reduce their losses simultaneously with the storage and release of water. In addition, hydrogels can be synthesized from natural raw materials to obtain biodegradable materials with a low or null residuality [18]. Examples of raw materials used for obtaining biodegradable-matrix hydrogels include starch, proteins, and cellulosic materials, among others. In particular, starch is one of the most abundant polysaccharides in nature, together with cellulose and chitin. Starch-based hydrogels have shown many advantages such as nontoxicity, biocompatibility, high biodegradability, efficient encapsulation, and prolonged release [6]. These have been classified into two main groups: (i) starch-based hydrogels resulting from the graft copolymerization of vinyl monomers onto starch in the presence of crosslinker, and (ii) starch-based hydrogels obtained by the direct crosslinking of starch chains among themselves or with other polymer chains [19]. The first group requires the use of petroleum-derivate polymers (i.e., acrylic acid); however, their use decreases the material’s biodegradability, limiting its application and causing the generation of persistent solid wastes. On the other hand, direct crosslinking can be induced using biodegradable species with both low and high molecular weight. Some examples are cellulose, collagen, and phosphorous compounds such as phosphorus oxychloride, sodium trimetaphosphate, and sodium tripolyphosphate; also, polyacids such as succinic acid and citric acid (CA) can be used [20,21,22,23]. 

Here, to promote the slow release of phosphorus, the storing of water, and the reduction in P requirements during fertilization operations, starch-based biodegradable hydrogels (SBHGs) were obtained from Cassava starch using CA as crosslinker, and these were characterized and evaluated to be applied in the controlled release of phosphorous, using two phosphorous sources: KH_2_PO_4_ and phosphate rock (phosphorite).

## 2. Results and Discussion

### 2.1. Hydrogel Formation and Characterization

**Figure 1:**
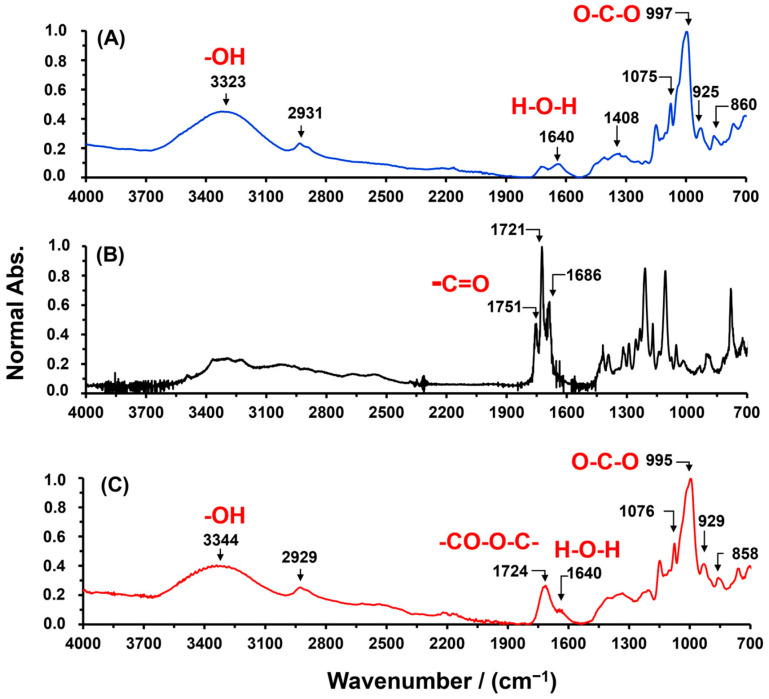
ATR-FITR spectra of (**A**) Cassava starch, (**B**) CA, and (**C**) SBHG(7%).

SBHG(7.0%) was obtained from an aqueous solution by the gelatinization of Cassava starch. The purpose of gelatinization was to open the starch granule and thus ease the reaction among the Cassava starch hydroxyl groups and carboxylic acid groups of CA. Thus, steric repulsion is reduced, and the crosslinking reaction is promoted. The spectra of FTIR of Cassava starch, CA and SBHG(7.0%) are shown in Figure 1. For Cassava starch, the bands in the range between 1700 cm^−1^ and 900 cm^−1^ are attributed to glucose (monomer units of amylose and amylopectin) [24,25]. Bands at 997, 925 and 860 cm^−1^ are attributed to the vibration of C–O–C bonds from carbohydrates, and the bands around 1075 cm^−1^ are assigned to C–O stretching. At 1408 cm^−1^, it is possible to see the symmetric deformation and scissoring of CH_2_ in the Cassava starch. Above 1700 cm^−1^, the stretching band from hydroxyl groups (3323 cm^−1^) and the stretching from C–H groups in the polymer chain (2931 cm^−1^) are identified. In addition, due to the high hydrophilicity, at 1640 cm^−1^, a band attributed to water molecules absorbed into the starch is observed [25,26,27]. 

For CA, characteristic bands of carbonyl group (C=O) from carboxylic groups are observed at approximately 1751, 1721, and 1686 cm^−1^; these correspond to stretching vibrations related to three acid groups [28,29]. On the other hand, as shown in Figure 1, for SBHG(7%), it is possible to identify the absorption band of the carbonyl group at 1724 cm^−1^, corresponding to the ester groups formed by the crosslinking reaction between the CA and Cassava starch. Similar bands in the region of 1720–1750 cm^−1^ were related to the stretching vibration of the carbonyl group in the spectra of starch crosslinked with CA by others researchers [20,30,31]. Therefore, the band at 1724 cm^−1^ was considered as evidence of the successful derivation of SBHG. In addition, in the fingerprint region, the bands at 1100–700 cm^−1^ did not show significant changes in their intensities or shifts in their wavenumbers, demonstrating that glucose units in Cassava starch maintain their chemical structure during the formation of the SBHG(7.0%). Additionally, the band around 1640 cm^−1^ evidences water entrapped inside the polymeric matrix, even after drying the material, which is consistent with the highly hydrophilic nature of the material. Similar results were obtained for the other compositions.

**Figure 2:**
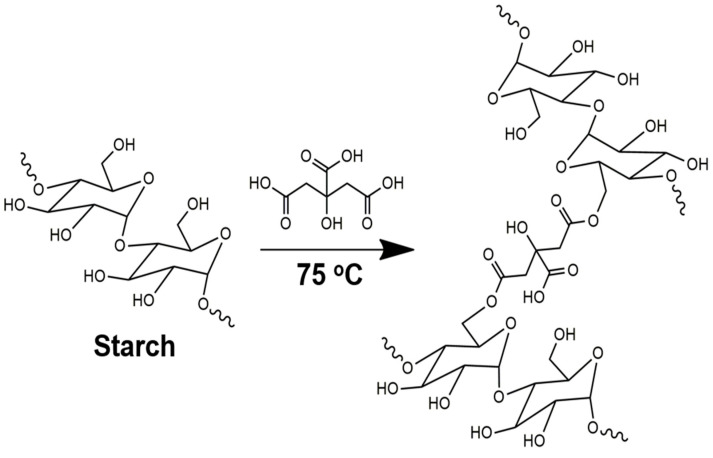
Illustration of the crosslinking of starch by Fischer esterification reaction with CA.

The Fisher esterification reaction between carboxyl groups of CA and the hydroxyl of Cassava starch is shown in Figure 2. The reaction is catalyzed by a low pH or by Lewis acids and drops in alkaline pH values due to the formation of carboxylates, which limit the reaction and decrease the esterification yield [29]. Thus, CA catalyzes the esterification, and, at the same time, it allows the crosslinking of starch chains (pH between 2 and 3). In this experiment, the use of another acid was not needed since the CA was sufficient to catalyze the reaction and form the SBHG. Thus, the making of a hydrogel is easy when using only three components: water, Cassava starch, and CA.

**Figure 3:**
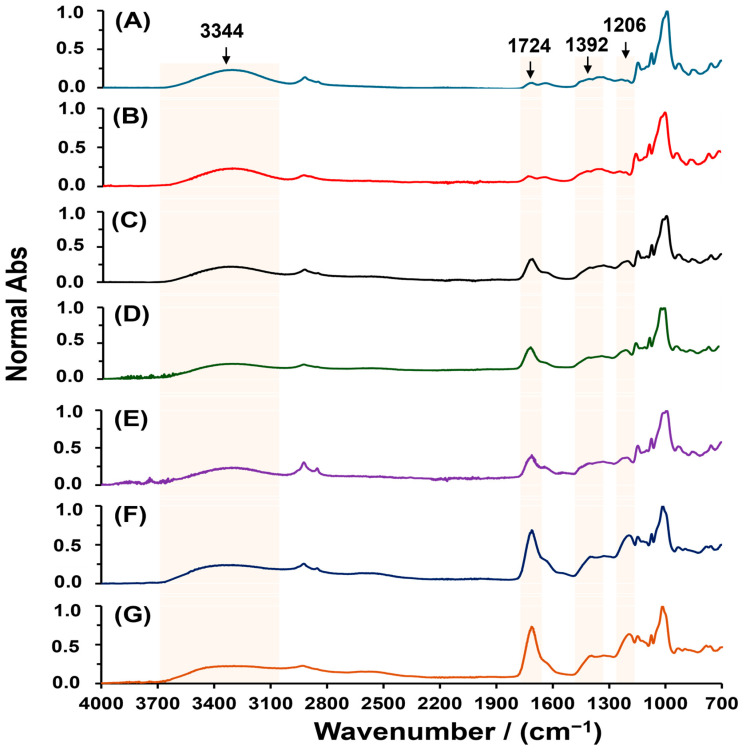
ATR-FITR spectra of dry hydrogels of Cassava starch crosslinked with various CA concentrations: 0.5 (**A**), 1.0 (**B**), 5.0 (**C**), 7.0 (**D**), 10.0 (**E**), 20.0 (**F**) and 40.0% *w*/*w* (**G**).

To further understand the formation process of SBHG(x), the effect of CA concentration (0.5 to 40.0% *w*/*w*) on esterification was monitored by ATR-FTIR. Figure 3 shows that the main bands change in the ATR infrared spectra of dry SBHG(x) as the CA concentration is increased. In all hydrogels, we observed a signal at 1724 cm^−1^ demonstrating the successful esterification reaction. In addition, as the CA concentration is increased, the intensity of the signal at 1724 cm^−1^ is increased, whereas the band related to hydroxyl groups is decreased (3344 cm^−1^); this behavior is associated with the conversion of hydroxyl groups to ester groups during esterification. The above is also evidenced by the increase in the intensity of asymmetric stretching vibration in ester groups (-CO–O–C-) at 1392 cm^−1^. A similar behavior was described by reference [32] during the esterification of amylose from corn starch and propionic anhydride. In addition, a new band at 1206 cm^−1^, corresponding to the C–H deformation vibration of CA, appears in the fingerprint region, which implies an increase in intensity as CA concentration is increased. From the previously described results, it can be concluded that crosslinking is promoted by the contents of CA, what allows the formation of a three-dimensional network of starch chains. 

**Figure 4:**
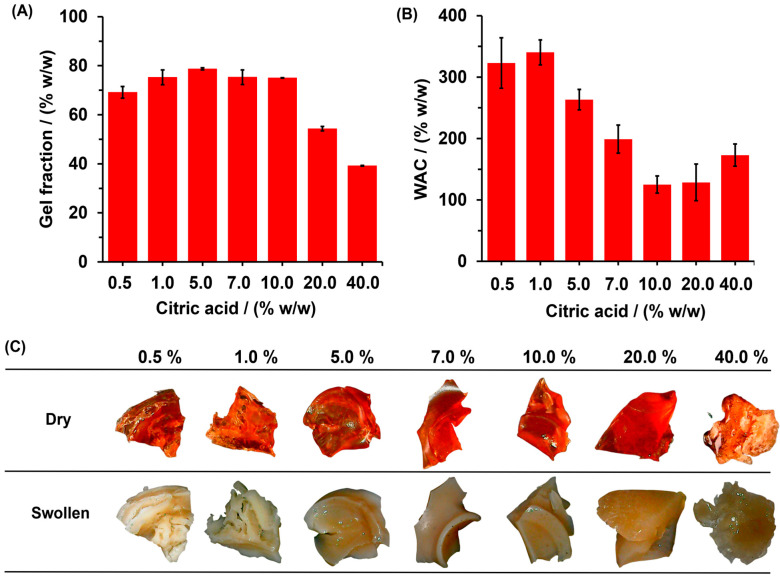
Effect of CA concentration on: (**A**) fraction gel and (**B**) WAC of SBHG. In addition, (**C**) digital photos of SBHG with various CA concentrations before (dry) and after the water-absorbing process (swollen).

On the other hand, the gel fraction can be understood as an indirect measurement of the percentage of polymer chain that is covalently linked and does not leach out from the hydrogel matrix. Figure 4A shows the effects of CA concentration on gel fraction percentage. The gel fraction increased from 69.1 ± 2.3% to 75.0 ± 0.09% as the CA concentration was increased from 0.5 to 10.0% of CA; after that, the gel fraction decreased to 39.1 ± 0.2 for hydrogel with 40.0% of CA. The results show that for all SBHG, the use of up to 20.0% of CA allows for a high degree of crosslinking between CA and Cassava starch. However, at higher concentration of CA (40.0%), a decrease in gel fraction was observed, which is probably due to the depolymerization reaction promoted by acid hydrolysis, which is a competitive reaction of esterification.

On the other hand, WAC decreased from 322.9 ± 41.0% to 125.1 ± 13.9% as CA concentration increased from 0.5 to 1.0%. For higher values of CA contents, WAC increased up to 172.9 ± 18.2%, corresponding to SBHG(40.0%) (Figure 4B). The decrease in WAC with the increase in the concentration of CA can be explained by the increase in crosslinikng and the subsequent decreasing of pore size and cavities in the polymer structure, which limits the diffusion of water towards the inside of the hydrogel. The above is consistent with previous studies, wherein a relationship between CA content and the degree of swelling was identified [30,31]. This behavior could be related to the material’s appearance as dry or swollen (Figure 4C). When the hydrogel is dry, hydrogels are hard to the touch and have a red-brown hue; however, when they are swollen, hydrogels are softened, increase in volume, and characterized by a whitish hue. After immersion in distilled water, swollen SBHG(7.0%) and SBHG(10.0%) showed less swelling compared to other hydrogels. This is an important characteristic of hydrogels for agricultural use, since it allows a reduction in the fertilizer release time. Thus, a lower rate of water diffusion will be related to lower fertilizer transference from the loading phase (hydrogel) to the surrounding environment, and from there to the site of action (plant).

**Figure 5:**
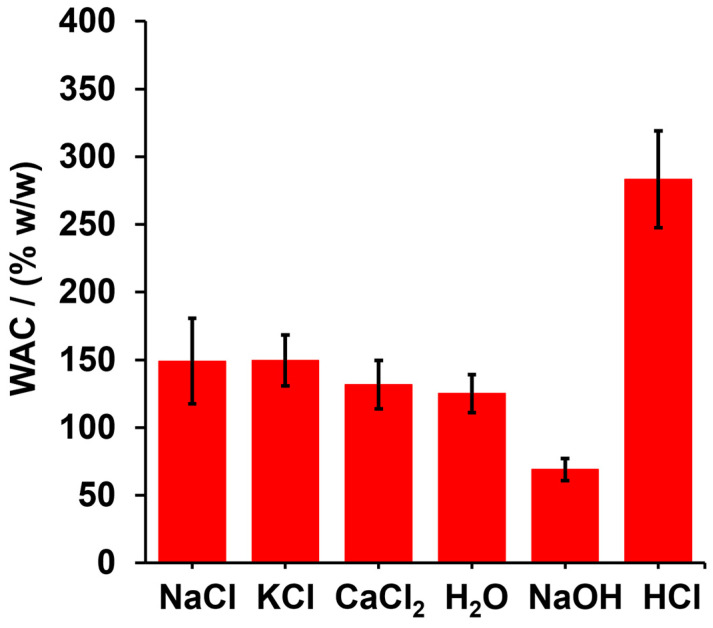
WAC of SBHG(10.0%) in different media containing 0.5% *w*/*v* of solute (NaCl, KCl, CaCl_2_, NaOH and HCl).

Though the swelling capacity of a hydrogel is expected to be influenced by medium composition, ionic strength, and pH as a result of changes in chemical and intermolecular interactions inside the hydrogel network, the results shows that there are no significant differences (*p* ≥ 0.05—paired student *t* test) when distilled water is used as the swelling medium in comparison with salt solutions (NaCl, KCl, and CaCl_2_); therefore, it is concluded that, at the tested concentration, the saline enviroment does not alter intermolecular interactions and does not produce significant changes in the hydrogel’s osmotic pressure, which is the driving force of hydrogel swelling. On the other hand, NaOH and HCl had opposing effects on WAC. Thus, whereas NaOH decreased the WAC, HCl increased it (see Figure 5). In both cases, significant differences in WAC were observed with respect to distilled water (*p* ≤ 0.05). In an alkaline medium, ester groups are more easily hydrolyzed, producing a decrease in crosslinking, and as a consequence, a part of the water contained in the hydrogel is drained by gravity. On the contrary, although hydrolysis can occur in acidic environments, its kinetic activity is relatively slower compared to alkaline hydrolysis, but also, the acid load inside the hydrogel can be decreased by the protonation of the carboxylic acid groups remaining in the polymer matrix. According to Le Chatelier’s principle, the esterefication reaction is controlled by chemical equilibrium, the acid enviroment being one of the factors that promote a shift in equilibrium towards the formation of the ester, with the subsequent decrease in protons in the surrounding environment.

**Figure 6:**
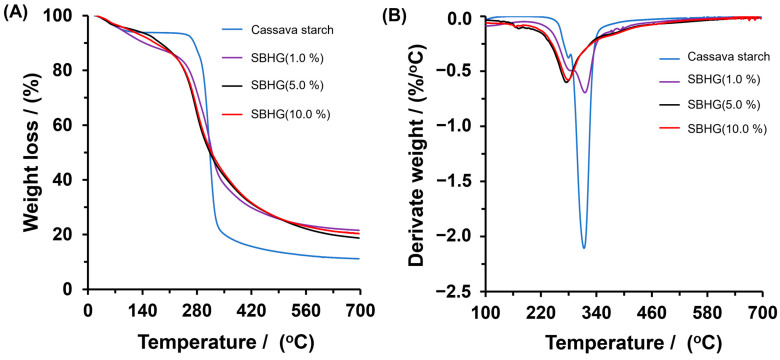
(**A**) TGA and (**B**) weight loss derivate curves of Cassava starch, SBHG(1.0%), SBHG(5.0%), and SBHG(10.0%).

To determine the effects of the CA concentration on the thermal stability of hydrogels, measurements of TGA were performed on starch, SBHG(1.0%), SBHG(5.0%) and SBHG(10.0%). The results are shown in Figure 6A,B. The starch showed two notable weight losses caused by thermal decomposition. The first one, which occered between 60 and 110 °C and invovled an 8% loss of mass, is attributed to the water absorbed and entrapped inside the starch sample, whereas the second one, at between 250 and 400 °C, corresponds to the thermal degradation of the starch’s backbone. The maximum mass loss was identified at 315 °C, according to the derivate curves (Figure 6B). 

The SBHGs showed behaviors similar to Cassava starch; however, the first stage, which ranged between 60 and 230 °C, was characterized by an almost linear trend of mass loss as the temperature increased, the mass loss being approximately 20% with respect to the mass of each samples. This stage is related to the elimination of water from the polymer matrix. The second stage started at 250 °C and finished at 400 °C, with its maximum mass loss decreasing as the CA concentration increased in the order of SBHG(1.0%) (at 311 °C) > SBHG(5.0%) (at 275 °C) > SBHG(10.0%) (at 274 °C). After that, at temperatures greater than 274 °C, the mass loss rate in hydrogels decreased, with a residue of about 25% *w*/*w* being obtained in the tested samples, ths being greater than that obtained from starch (~12.0%), which indicates a very much improved thermal resistance for SBHG compared to Cassava starch. A similar behavior was observed by reference [33], who concluded that CA increases the resistance to thermal degradation in hydrogels made from CA and carboxymethyl sago starch.

### 2.2. In Vitro Studies of Phosphorus Release

**Figure 7:**
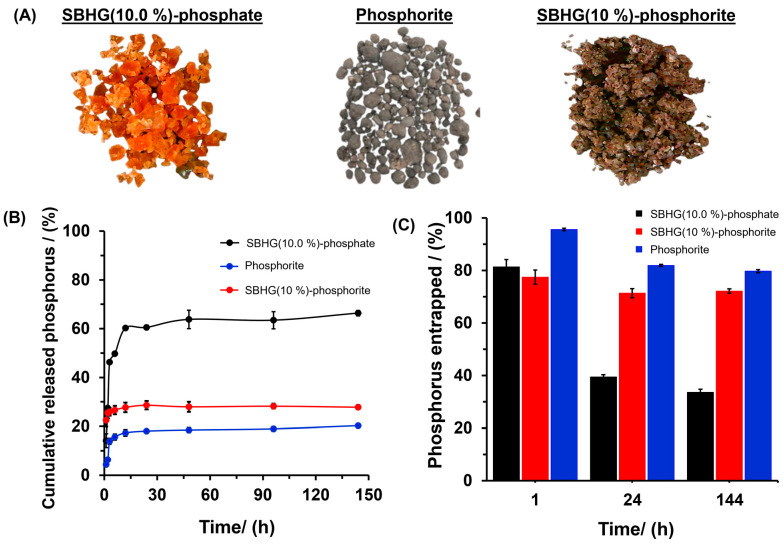
(**A**) Digital photos of materials containing P, and (**B**) in vitro release profiles of phosphorus and (**C**) phosphorus entrapped after 1, 24, and 144 h in the release experiment on SBHG(10.0%)-phosphate, phosphorite (without ground), and SBHG(10%)-phosphorite.

SBHG(10.0%) showed the lowest WAC, indicating that hydrogels with 10% *w*/*w* of CA are characterized by the greatest crosslinking degree within the range of concentrations evaluated. Thus, SBHG(10.0%) was loaded with phosphorus in order to evaluate its release. Thus, the content of phosphorous comprised the sum of phosphorus derived from KH_2_PO_4_ (82.5 ± 1.6 mg g^−1^) and from phosphorite (68.6 ± 0.4 mg g^−1^). The SBHG(10.0%)-P showed a visual appearance similar to that of SBHG(10.0%) (Figure 4C and Figure 7A). However, hydrogels containing phosphorite showed a grayish hue. Phosphorite (Figure 7A) is a P-rich rock, normally composed of carbonate hydroxyl fluorapatite (Ca_10_(PO_4_CO_3_)_6_F_2-3_) [34], which provides PO_4_^3−^ ions to the crops when it is used as a fertilizer. The phosphorite used in this research contained 169.5 ± 0.32 mg g^−1^ of phosphorus.

The phosphorus release profile from SBHG(10.0%)-P showed a rapid and progressive release, reaching approximately 80% in the first 15 h (Figure 7B), followed by a slight increase up to 88% in 144 h (i.e., a variation of only 8 percentage units over almost ten times as long). When phosphorite was used, the phosphorus released from hydrogel showed a similar behavior to that observed for SBHG(10.0%)-P, releasing almost 73% in the first 15 h until reaching a constant value independent of time. On the other hand, the phosphorus release time decreased for the phosphorite contained in SBHG(10.0%), which delivering around 48% of its phosphorus in the first 15 h, a rate that gradually increased until approximately 50% in 144 h. The results indicate that the phosphorite entrapped in SBHG(10.0%) was delivered slower than the rate observed for other tested materials, which is desirable, since it will aid in creating phosphorus slow-release systems. In addition, according to Figure 7C, phosphorus concentration deacreses as time is increased for all the materials; however, SBHG(10.0%)-phosphorite showed the greatest level of nutrient release after 144 h. 

Four kinetic models (zero-order, first-order, Higuchi, and Power law) were used to evaluate the mechanism of phosphorus release from hydrogels (see Table 1). The Higuchi model showed higher correlation coefficients (R^2^) for both SBHG(10.0%)-P and SBHG(10.0%)-phosphorite. This model describes the diffusion of a solute from a matrix with different geometric dimensions and porous systems, wherein the diffusion process occurs via matrix swelling, promoting the diffusion of solute at a constant rate [35,36]. Thus, in both cases, it is possible to identify three stages of the absorption–release processes (loading–unloading): (i) When the hydrogel is introduced into an aqueous solution, due to the difference in water potential inside the hydrogel with respect to the surrounding medium, the solvent and the solutes flow into the interior of the hydrogel, leading to the incorporation of water and solutes, and subsequent matrix swelling. Although this first stage corresponds to the loading of the material and not to its release, it is important because it suggests that after swelling the hydrogel is not in equilibrium, but rather that an adsorption processes has taken place inside it. Flow-mediated incorporation is the result of the dry condition of the material, so diffusion and adsorption are not significant. However, inside the hydrogel, the forces of interaction of the matrix with the medium are different; in principle, the medium is more viscous, which makes diffusive processes difficult, but they are not eliminated. (ii) During the release experiments, the swollen hydrogels are introduced into water, creating a heterogeneous system with two characteristics. The first relates to its two zones, an external zone (water) and an internal zone (a gelled fertilizer solution), separated by a common interface. In this conext, the change in hydric potential between the different states is zero; therefore, it is expected that water flow does not occur in any direction. However, the phosphate ions contained in the polymer matrix can diffuse from the interior of the hydrogel to the surrounding medium (i.e., the chemical potential of the phosphate ion is higher in the hydrogel compared to pure water). Consequently, the first stage is expected to be controlled by diffusion. (iii) In addition to diffusion, dissolution appears to be a determinative process when phosphorite is present. Unlike phosphate ions, which are found in the solution, phosphorite is dispersed in particulate form. Therefore, the viscosity of the surrounding medium reduces the availability of water to promote dissolution. Inside the hydrogel, water molecules interact strongly with the matrix, their mobility is reduced, and the hydration of the phosphotite particles is strongly limited. Consequently, an appreciable fraction of the phosphorite remains retained inside the hydrogel matrix. 

The above is consistent with Higuchi’s model. It can be seen that for SBHG(10.0%), the Higuchi’s rate constant was higher when hydrogel was loaded with phosphate than with phosphorite (see Figure 7A). This result shows that SBHG(10.0%)-phosphorite is a promising material that can be used to provide phosphorus gradually in crop production; according to the literature, phosphorus showed a faster release profile from polysaccharide hydrogel. For instance, reference [37] reported a release profile lasting around 40 min for phosphorus derived from cellulose hydrogel. Similar results were reported for monoammonium phosphate fertilizer composed of a sodium carboxymethyl cellulose/hydroxyethyl cellulose blend filled with 5% spherical regenerated cellulose particles, where ~90% of the fertilizer encapsulated was released over 3 h [38].

**Figure 8:**
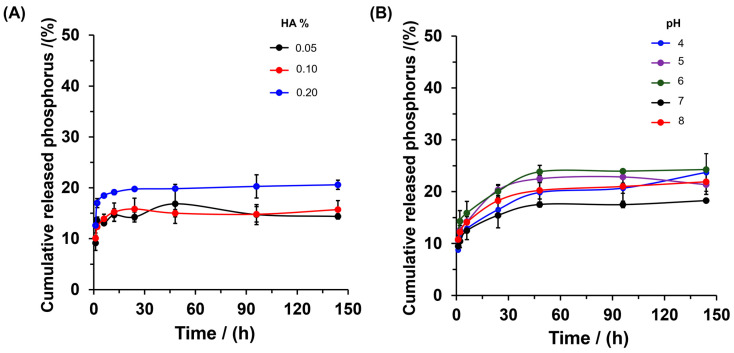
In vitro release profiles of phosphorus from SBHG(10.0%)-phosphorite at different concentrations of HA (**A**) and different pH values (**B**). the NaOH 1.0 M solution in deionized water was used as the release medium for HA, and the results are represented as the mean SD of triplicate experiments.

Previous studies have demonstrated that the addition of organic matter such as humic acid (HA) and fulvic acid produces a positive effect on soils, due to increasing phosphorus mobility and enhanced crop yield [39,40]. Therefore, to evaluate the effect of HA concentration on the delivery behavior of phosphorus from SBHG(10.0%)-phosphorite, the phosphorus release kinetic was determined up to 144 h (Figure 8). In HA 0.05% and 0.10%, after 144 h, the SBHG(10.0%)-phosphorite delivered between 35% and 40% of phosphorus, which is lower than the level of phosphorus released from distilled water (~50%). However, with 0.2% HA, the hydrogel delivered approximately 55% of the phosphorus at 144 h. Thus, the results suggest that a low HA concentration (0.05 and 0.10%) decreases the capacity for phosphorus delivery, whereas at higher concentrations (0.2% of HA), we see a significant enhancement in the release of phosphorus. These results can be attributed to the chemical interaction between the HA molecules and phosphate ions. In an alkaline medium (used to ensure solubility of HA), HA molecules present electrostatic repulsion produced by the deprotonated phenolic and carboxylic groups [41]. 

Thus, at low HA concentrations, an unfavorable chemical interaction between PO_4_^3−^ ions and HA is expected; as a consequence, the PO_4_^3−^ diffusion rate is decreased. However, the aggregation of HA in solution could decrease the electrostatic repulsion between PO_4_^3−^ ions and HA; thus, by increasing the favorable interaction between PO_4_^3−^ ions and HA, we see a transfer of PO_4_^3−^ from the solid phase to the alkaline aqueous solution. These results are in agreement with those reported by reference [42], who found that HA affects the adsorption of PO_4_^3−^ on goethite-coated silica sand via batch isotherms and column experiments under steady-state flow conditions. However, the presence of HA could produce a positive effect due to the increase in PO_4_^3−^ release, favoring phosphorus’ contribution to plant nutrition. On the other hand, the effectof pH on phosphorus delivery was evaluated for SBHG(10.0%)-phosphorite (Figure 8B). It was found that the hydrogel exhibited a tendency to increase phosphorus release in an acidic medium (pH 4–6). As was demonstrated, in an acidic medium, the material showed higher WAC (Figure 5), increasing the content of medium released from the hydrogel. Consequently, under this condition, the solubility of the phosphorite rock entrapped in the hydrogel increased, thereby increasing the nutrient diffusion rate. 

The slow release of P from SBHG(10.0%) described above offers an alternative means to provide the nutritional requirements of crops while reducing the negative impact of conventional P fertilizers. Additionally, SBHG could offer an alternative way to overcome the limitations of the large-scale production and commercialization of hydrogel nutrient carriers. For example, PUSA hydrogel, a cellulose-graft anionic polyacrylate superabsorbent polymer applied to encourage water release during dry periods (without fertilizer), showed a higher cost (USD 14–18/kg) with application levels of 2.5–3.0 kg/ha. The high cost of production and manufacturing limited its application in crop production. ALSTA hydrogel, on the other hand, is a more economical alternative (USD 8–9/kg) for use in nutrient release, and is based on potassium polyacrylate, which can reduce fertilizer use [43]. Thus, the low-cost production of SBHG(10.0%), owed to it being composed of only Cassava starch and CA, presents advantages related to commercial viability. Additionally, SBHG is a green, biodegradable material, thus overcoming the biodegradability issue of hydrogels made from synthetic polymers. However, even in this work, the mechanical properties of SBHG were not measured, and its mechanical strength could be limited upon swelling, characteristic of biopolymer-based hydrogels [44]. New experiments should be performed to understand the behavior of SBHG in different soils and its effects on nutrient release to promote plant growth.

## 3. Conclusions

An SBHG was synthesized and applied to encourage the slow release of phosphorus through a low-cost and simple method; thus, from the results, different aspects can be inferred. First, the crosslinking of starch with CA is easily assessed by ATR-FTIR and swelling tests. This is occurs at different concentrations of CA from 0.5 to 40%. In addition, it was shown that the degree of starch crosslinking reduces the WAC and improves the thermal resistance of hydrogels. On the other hand, the release of phosphorus from SBHG(10.0%)-phosphorite was initially rapid, followed by a slow, sustained and continuous release stage until 144 h of immersion. In addition, it was found that the HA concentration promotes the delivery of the nutrient from a hydrogel (SBHG(10.0%) loaded with phosphorite) via the favorable chemical interaction between HA aggregates and PO_4_^3−^ ions. In summary, this study has demonstrated that the combined use of SBHG and HA can be useful in promoting the steady and sustained release of phosphorus, which could have high potential utility in the slow release of nutrients in crops; however, the potential use of this hydrogel in plant growth, the effects of field conditions, and its residual time in the soil must be evaluated.

## 4. Materials and Methods

### 4.1. Materials

Cassava starch and phosphorite were obtained from a local market in Cali, Colombia. CA (C_6_H_8_O_7_), potassium dihydrogen phosphate (KH_2_PO_4_), sodium chloride (NaCl), potassium chloride (KCl), sodium hydroxide (NaOH), calcium chloride (CaCl_2_), and hydrochloric acid (HCl) were purchased from Sigma-Aldrich (St. Louis, MA, USA).

### 4.2. Preparation of SBHGs

SBHGs were formed as follows: A mixture of 6.0 g of starch and 50 g of distilled water was combined, stirred, and heated at 75 °C for 30 min until starch gelatinization was achieved. A predetermined quantity of CA was added to obtain a CA concentration of 0.5% *w*/*w*. The mixture was continuously stirred for 1 h at 75 °C. Later, the material was dried at 60 °C for 24 h, washed with distilled water to remove the unreacted CA, and dried again at 60 °C for 72 h. The same procedure was used to form several SBHGs named SBHG(x), where x is the concentration of CA used (x = 0.5, 1.0, 5.0, 7.0, 10.0, 20.0 and 40.0%).

### 4.3. Gel Fraction

The gel fraction was determined following the research reported by reference [45], with some modifications. To determine the gel fraction, the pre-weighed SBHG(x) was taken in a beaker and immersed in a sufficient amount of distilled water for 24 h. After this time, the water was removed, and the material was dried in an oven at 50 °C until the weight was constant. The gel fraction was calculated using Equation (1):

(1) $$Gel\;fraction(\%)=\left(\frac{W_{f}}{W_{0}}\right)$$

where W_f_ and W_0_ are the final dried weight and the initial dried weight, respectively. Note that the gel fraction is a measure of the degree of effectiveness of the starch crosslinking process, performed through esterification with CA.

### 4.4. Swelling Studies

The water absorption capacity (WAC) was determined by the tea bag method [46]. A mass of SBHG(x) was added to a tea bag that had previously been weighed. Later, the tea bag containing the hydrogel was immersed in water for 24 h. After that, the bag with the hydrogel was hung up for 15 min to remove the excess water via gravity, and weighed. The WAC was calculated using Equation (2). A blank experiment was performed using only tea bag.

(2) $$WAC=\frac{w_{2}-(w_{1}+w_{0})}{w_{1}}$$

where $w_{0}$, $w_{1}$ and $w_{2}$ are the masses of the tea bag, the SBHG(x) sample, and the tea bag containing SBHG(x), respectively. These experiments were performed in triplicate. 

### 4.5. Physicochemical and Morphological Characterization of SBHG(x)

The SBHG(x) was characterized by Fourier-transformed infrared spectroscopy using the technique of attenuated total reflectance (ATR-FTIR, IR-Affinity-1, Shimadzu, Kyoto, Japan). The spectra were recorded to verify the esterification reaction. The data were acquired from 4000 to 700 cm^−1^ at a rate of 2 cm^−1^ per point, with 20 scans per sample. Thermal analysis was performed using a thermogravimetric analyzer (TGA, SDT-Q600, TA instrument, New Castle, DE, USA). Thus, powdered starch and SBHG(x) were weighed (5–10 mg) and heated from room temperature to 650 °C at a constant heating rate of 10 °C/min under a nitrogen flow of 20 mL per min. The results were evaluated by considering the first-order derivative of the raw weight loss thermogram. 

### 4.6. Preparation of Phosphorous-Loaded SBHG(x)

SBHG(10.0%) was produced via the methodology described in Section 4.2, with the addition of KH_2_PO_4_ and phosphorite (previously ground and sieved to 250 μm) to form SBHG with phosphate (10% *w*/*w*) and SBHG with phosphorite (10.0% *w*/*w*). Samples of both materials were digested with concentrated HCl, and the phosphorus concentrations were quantified by UV-Vis spectroscopy (DU-8800DS, Chongqing Drawell Instrument Co., Ltd., Chongqing, China) using a calibration curve via the molybdovanadate method reported by NTC 6259 [47].

### 4.7. Phosphorous Release Kinetics—In Vitro Study

In separate experiments, 0.5 g samples of SBHG(10.0%) loaded with phosphate, and SBHG(10.0%) loaded with phosphorite and phosphorite, were placed in a tea bag. The phosphorous concentration was evaluated in the solution as a function of time. The samples were kept under conditions of constant stirring at 100 rpm, using deionized water as the release medium. Aliquots (0.5 mL) of the resulting solution were taken at specified time intervals over six days, and immediately replaced with fresh medium. Additionally, following the methodology described above, an in vitro release study was performed using SBHG(10.0%)-phosphorite at different HA concentrations (0.05, 0.1, and 0.2% *w*/*v*) and pH values (4, 5, 6, 7, and 8). To determine the effect of HA, NaOH 1.0 M solution in deionized water was used as the release medium. The experiments were carried out in triplicate. 

The release mechanism was evaluated using four kinetic models: zero-order, first-order, Higuchi, and Power law. These can be represented via the following Equations (3)–(6):

(3) $$\mathrm{Zero-order}\;\mathrm{model}:\;M_{t}/M_{\infty }=k_{0}t$$

(4) $$\mathrm{First-order}\;\mathrm{model}:\;M_{t}/M_{\infty }=1-e^{\left(-k_{1}t\right)}$$

(5) $$\mathrm{Higuchi}\;\mathrm{model}:\;M_{t}/M_{\infty }=k_{H}t^{\frac{1}{2}}$$

(6) $$\mathrm{Power}\;\mathrm{law}\;\mathrm{model}:\;M_{t}/M_{\infty }=kt^{n}$$

where $M_{t}/M_{\infty }$ is the drug fraction released at time t; $k_{0}$, $k_{1}$, $k_{H}$ and k represent the zero-order release constant, first-order release constant, Higuchi constant and Power law constant, respectively [48]. The release constants and the parameters of the models were derived from the fitting of the experimental release data.

## Figures and Tables

**Table 1:** Kinetics parameters of phosphorus release from the SBHG(10.0%) loaded with phosphate and phosphorite.

Parameters of Model		Load	
		Phosphate	Phosphorite
Zero-order	$k_{0}\;(1\times 10^{-2}h^{-1})$	9.71	6.89
	R^2^	0.844	0.922
First-order	$k_{1}$ $\left(1\times 10^{-2}h^{-1}\right)$	0.19	0.15
	R^2^	0.728	0.705
Higuchi	$k_{H}$ $\left(1\times 10^{-2}h^{-1}\right)$	0.36	0.25
	R^2^	0.929	0.973
Power law	k $\left(1\times 10^{-2}h^{-1}\right)$	2.76	1.85
	R^2^	0.928	0.911
	n	0.62	0.50

k_0_, k_1_ “k_H_” and “k” represent the apparent rate constants of respective models, and n is the release exponent of the Power law model.

## Data Availability

Data are contained within the article.
